# Gut microbiota modulation accounts for the neuroprotective properties of anthocyanins

**DOI:** 10.1038/s41598-018-29744-5

**Published:** 2018-07-27

**Authors:** Cláudia Marques, Iva Fernandes, Manuela Meireles, Ana Faria, Jeremy P. E. Spencer, Nuno Mateus, Conceição Calhau

**Affiliations:** 1 CINTESIS - Centre for Research in Health Technologies and Information Systems, Porto, Portugal; 2 Nutrition & Metabolism, NOVA Medical School, Faculdade de Ciências Médicas, Universidade Nova de Lisboa, Lisboa, Portugal; 3 REQUIMTE/LAQV, Department of Chemistry and Biochemistry, Faculty of Sciences, University of Porto, Porto, Portugal; 4 ESSUAlg - School of Health, University of Algarve, Faro, Portugal; 5 Comprehensive Health Research Centre, Universidade Nova de Lisboa, Lisboa, Portugal; 6 Hugh Sinclair Unit for Human Nutrition, School of Chemistry, Food and Pharmacy, University of Reading, Reading, UK

## Abstract

High-fat (HF) diets are thought to disrupt the profile of the gut microbiota in a manner that may contribute to the neuroinflammation and neurobehavioral changes observed in obesity. Accordingly, we hypothesize that by preventing HF-diet induced dysbiosis it is possible to prevent neuroinflammation and the consequent neurological disorders. Anthocyanins are flavonoids found in berries that exhibit anti-neuroinflammatory properties in the context of obesity. Here, we demonstrate that the blackberry anthocyanin-rich extract (BE) can modulate gut microbiota composition and counteract some of the features of HF-diet induced dysbiosis. In addition, we show that the modifications in gut microbial environment are partially linked with the anti-neuroinflammatory properties of BE. Through fecal metabolome analysis, we unravel the mechanism by which BE participates in the bilateral communication between the gut and the brain. BE alters host tryptophan metabolism, increasing the production of the neuroprotective metabolite kynurenic acid. These findings strongly suggest that dietary manipulation of the gut microbiota with anthocyanins can attenuate the neurologic complications of obesity, thus expanding the classification of psychobiotics to anthocyanins.

## Introduction

The incidence of neurodegenerative diseases, such as Alzheimer’s and Parkinson’s diseases has been increasing as global population gets older. The World Health Organization (WHO) predicts that by 2040, neurodegenerative diseases will overtake cancer to become the second leading cause of death after cardiovascular disease^1^. Accumulating evidence suggests that neurodegeneration occurs, in part, because the neuronal environment is disturbed in a cascade of processes collectively called neuroinflammation^2^. Neuroinflammation is also central in other psychiatry disorders such as anxiety and depression which are often associated with obesity^3,4^. Neuroinflammatory processes are profoundly modulated by peripheral inflammatory stimuli, especially those coming from the gut microbiota^5–7^. In this regard, both central nervous system (CNS) and gut microbiota offer legitimate targets for novel therapeutic strategies aiming to treat the rising burden of neurodegenerative and neuropsychiatry disorders. Indeed, live microorganisms that produce a health benefit in patients suffering from psychiatric illness (psychobiotics) have been proposed as psychotropic drugs^8^. Recently, the definition of psychobiotics have been extended to also encompass prebiotics^9^.

Flavonoids are a class of polyphenolic compounds that have been proposed as novel agents capable of attenuating neurodegenerative pathology^10^. Data suggest that anthocyanins (often consumed in higher amounts than other flavonoids^11^) interact with neurons and microglia, facilitating synaptic connectivity under both normal and pathologic conditions^12^. In addition, as recently demonstrated, anthocyanins are able to attenuate the negative impact of high-fat (HF) diets on neuroinflammation^13^, although their precise mechanism of action is unclear. One possibility is that they may exert their effects through modulation of the gut microbiota^14^.

The aim of the present study was to test the hypothesis that anthocyanins modulate gut microbiota composition and prevent HF diet-induced dysbiosis. After confirming these assumptions and given the bilateral connectivity that exists between gut and brain (the gut-brain axis)^15,16^, further investigations were carried out to study whether the anti-neuroinflammatory properties of anthocyanins were related to the gut microbiota changes that these compounds bring about.

## Results

### Blackberry anthocyanin-rich extract (BE) modulate gut-microbiota composition and counteract some of the features of HF diet-induced dysbiosis

We compared the gut microbial community of Wistar rats fed either with standard (C) or high-fat (HF) diet and supplemented with BE by sequencing the V3-V4 regions of the 16S rRNA gene.

**Figure 1:**
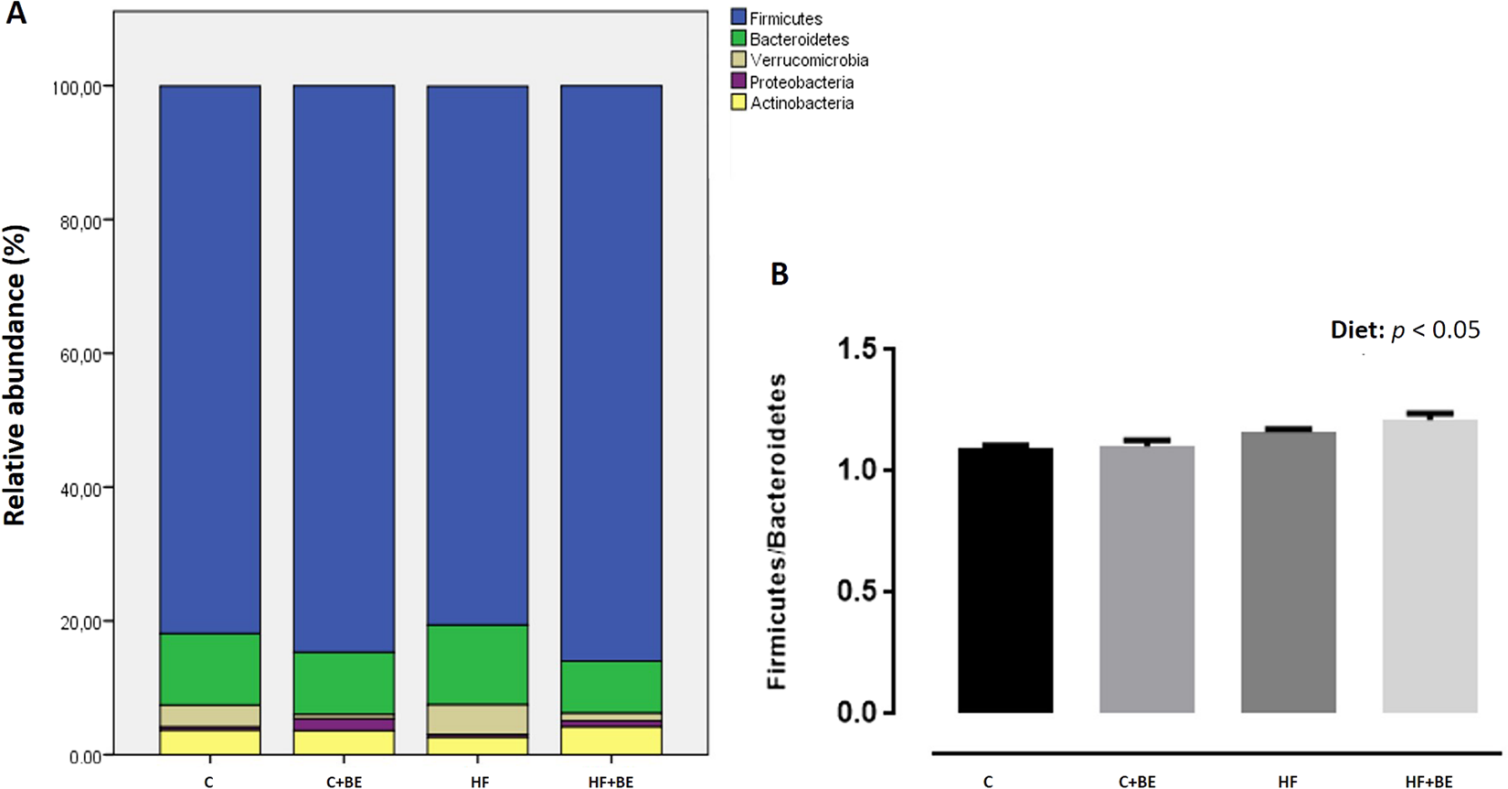
(**A**) Major bacterial phyla in the gut microbiota of rats after 17 weeks of high-fat (HF) feeding and anthocyanin-rich blackberry extract (BE) supplementation. Bars represent the average of each phylum relative abundance in the 4 different diet groups. Each phylum is represented by a different color (n = 5–6 rats per group). (**B**) Firmicutes to Bacteroidetes ratio among groups. This ratio was calculated by dividing the number of copies of Firmicutes by the number of copies of Bacteroidetes quantified by real-time PCR. Values are expressed as mean ± SEM (n = 5–6 rats per group).

The gut microbiota of the animals was dominantly constituted by five phyla: Firmicutes, Bacteroidetes, Actinobacteria, Verrucomicrobia and Proteobacteria (Fig. 1A). The Firmicutes to Bacteroidetes ratio was significantly increased in HF diet groups (*p* > 0.05) (Fig. 1B).

**Figure 2:**
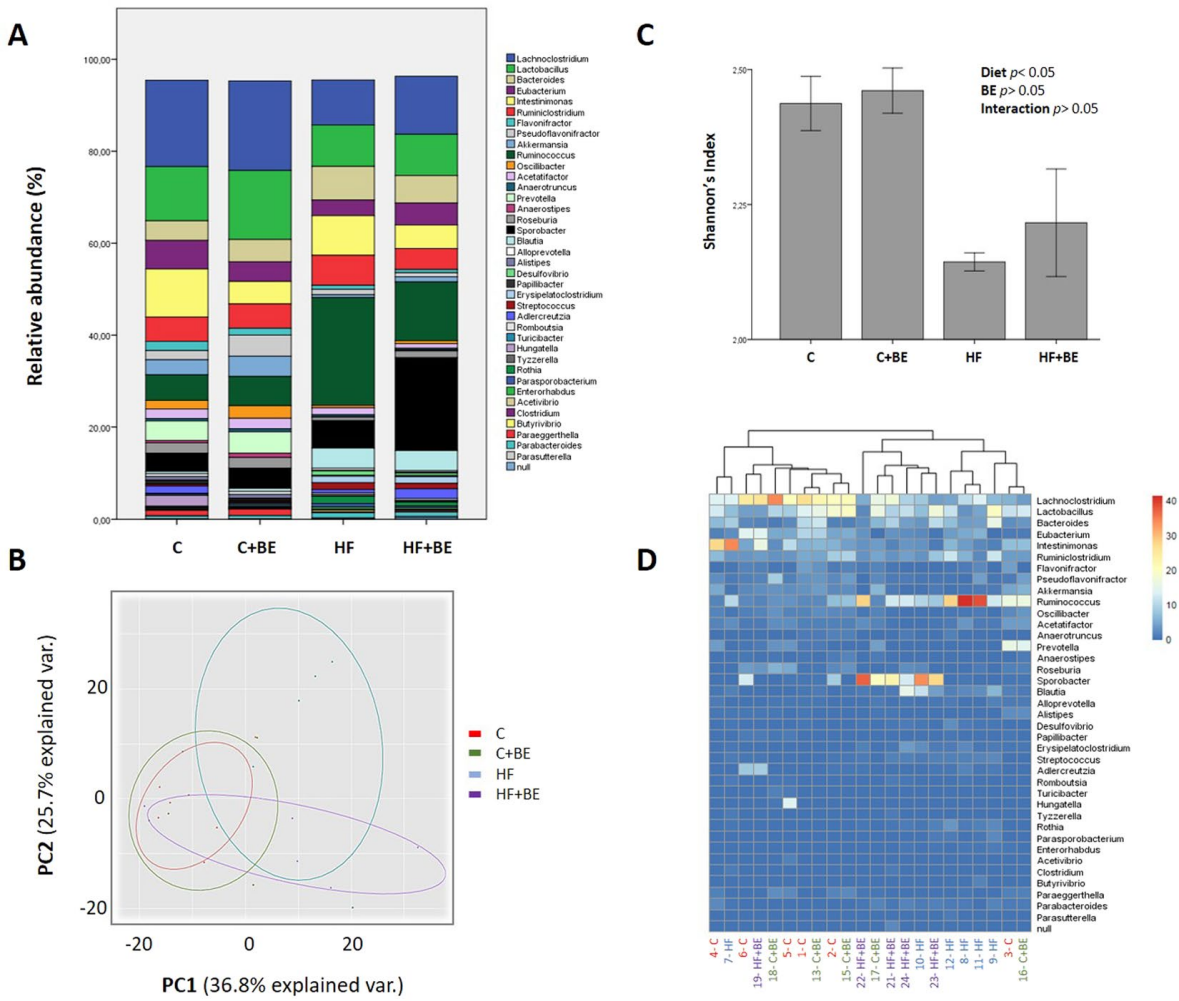
Gut microbiota composition at the genus level among groups (n = 5–6 per group). (**A**) Relative abundance of gut bacterial genera. Bars represent the average of each genus relative abundance in the 4 different diet groups. Each genus is represented by a different color. (**B**) Shannon’s diversity index among groups. Values are expressed as mean ± SEM. (**C**) Gut bacterial genera were clustered using principal component analysis (PCoA). Results are plotted according to the first two principle components, which explain 36.8% (PC1) and 25.7% (PC2) of the variation in gut microbial composition (at genus level) between samples. Each point represents one sample and each diet group is denoted by a different color. Circles combine samples from the same diet group by their respective 95% confidence interval ellipse. (**D**) Heatmap and hierarchical clustering of the relative abundance of gut bacterial genera. Rows correspond to operational taxonomic units (OTUs) and columns represent the animals of the 4 different diet groups.

Despite no differences observed at phylum level, at genus level, major differences were noted (Fig. 2A).

Results illustrated in Fig. 2A indicate the genus level distribution for the 4 diet groups. After 17 weeks of HF feeding, the profile of the gut microbiota of HF fed animals was significantly altered in comparison to the gut microbiota of control fed animals. Notably, bacteria belonging to the Firmicutes phylum such as *Rumminococcus*, *Blautia*, *Erysipelatoclostridium*, *Streptococcus* and Parasporobacterium were more abundant in HF fed animals (*p* < 0.05), whereas *Lachnoclostridium*, *Pseudoflavonifractor*, *Oscillibacter* and *Anaerostipes* (also Firmicutes) were more abundant in the animals of C group (*p* < 0.05) (Fig. 2A). In addition, HF diet decreased the abundance of *Akkermansia*, *Prevotella* and *Paraeggerthella* (*p* < 0.05), whereas it increased the abundance of *Desulfovibrio*, *Rothia* and *Enterohabdus* (*p* < 0.05) (Fig. 2A).

BE feeding induced several modifications in the gut microbiota composition of animals. Specifically, BE increased *Pseudoflavonifractor* when the animals were fed with C diet and increased *Oscillobacter* independently of the diet fat content (*p* < 0.05) (Fig. 2A). Moreover, BE struggled to recover the gut microbiota’s diversity when the animals were challenged with HF diet, as indicated by Shannon’s richness index (Fig. 2C).

To reduce the number of variables, a principal coordinate analysis (PCoA) was performed. The gut microbiota communities from the four different groups (C, BE, HF and HFBE) were grouped into four different clusters (Fig. 2C). HF fed rats had a distinct gut microbiota that clustered separately from C and BE rats. Nevertheless, the gut microbiota communities of the HF + BE animals were also differentiated from those of HF rats (Fig. 2C). The reduction of *Rumminococcu*s and the prevalence of *Sporobacter* were the main features that justified the segregation of HF + BE from HF group (Fig. 2D).

In summary, our data indicate that HF feeding strongly affected the composition of the gut microbiota and that BE can prevent some of the features of HF-diet induced dysbiosis (*e*.*g*. the increase of *Rumminococcu*s and the decrease of *Oscillobacter*).

### Changes in gut bacterial genera induced by BE are correlated with anti-neuroinflammatory properties

After characterizing the gut microbiota of the animals supplemented with BE, we sought to investigate if the protection against neuroinflammation, previously evaluated by our group, was associated with the changes observed in the gut bacterial genera of these animals.

**Figure 3:**
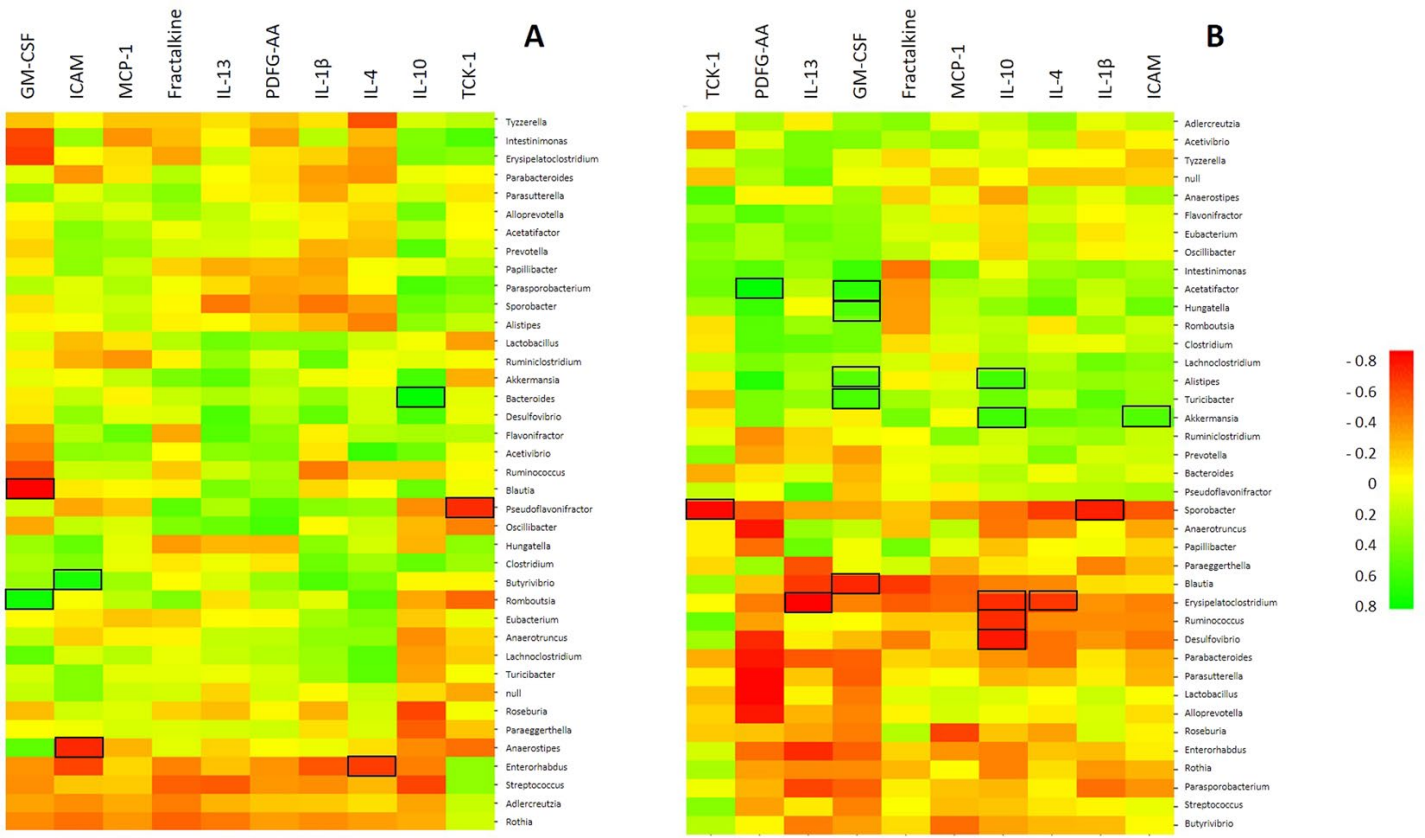
Heatmap of Spearman’s correlation test between gut bacterial genera and neuroinflammatory markers measured in hippocampus, (**A**) in the animals fed with standard diet (C and BE groups) and (**B**) in high-fat fed animals (HF and HFBE groups). Green color indicates a positive correlation while red color indicates a negative correlation. Squared cells represent correlations with statistical significance (*p* < 0.05).

In a previous work, we showed that BE consumption decreased TCK-1 expression in rat’s hippocampus whereas fractalkine expression increased^13^. Here, we found that TCK-1 was negatively correlated with *Pseudoflavonifractor* and *Sporobacter* when the animals were fed with C and HF diet, respectively (*p* < 0.05) (Fig. 3A,B). Interestingly, these bacterial genera were those increased by BE in C and HF diet groups, respectively (Fig. 2A). However, fractalkine, a chemokine particularly important in the crosstalk between neurons and microglia, was not correlated with any bacterial genus despite being upregulated in BE supplemented rat’s hippocampus (Fig. 3A,B).

### BE modulate CNS inflammation via the microbial metabolites of tryptophan

**Figure 4:**
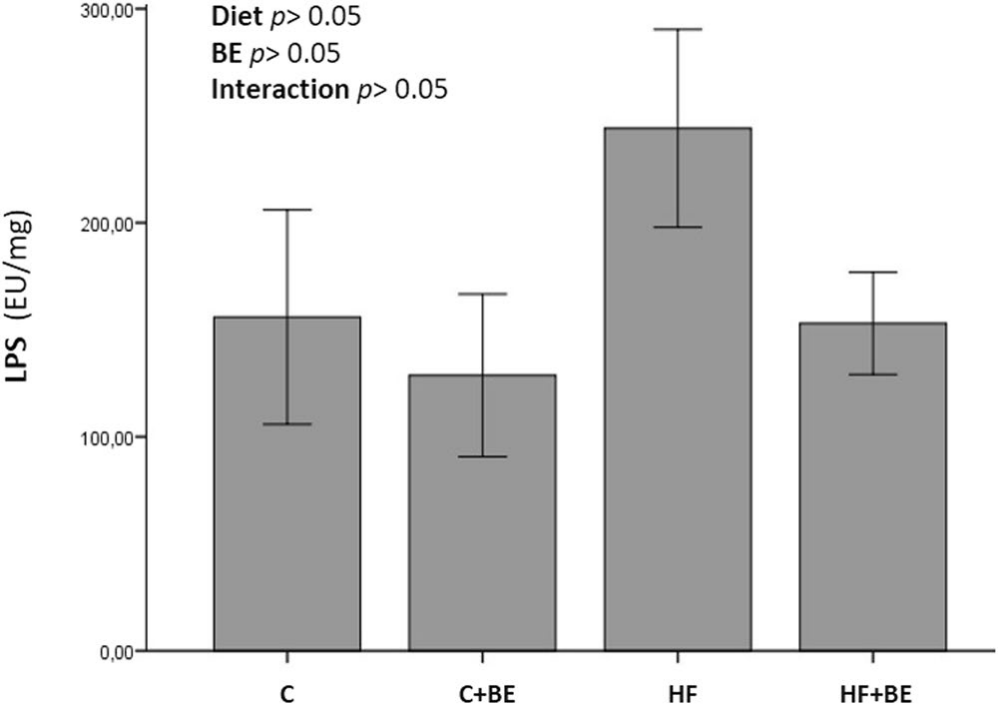
Fecal LPS concentrations. Values are expressed as mean ± SEM (n = 5–6 rats per group).

As a component of the gram-negative bacteria and a trigger of the inflammatory response, lipopolysaccharide (LPS) was measured in the fecal samples of all animals. Although fecal LPS levels were not statistically different between groups (*p* > 0.05), a noticeable increase in LPS levels was observed in the fecal samples of the animals of HF diet group (Fig. 4). In contrast, in HF + BE animals, fecal LPS was similar to that measured in control fed animals, probably as a reflection of the gut microbiota modifications induced by BE (Fig. 4).

**Figure 5:**
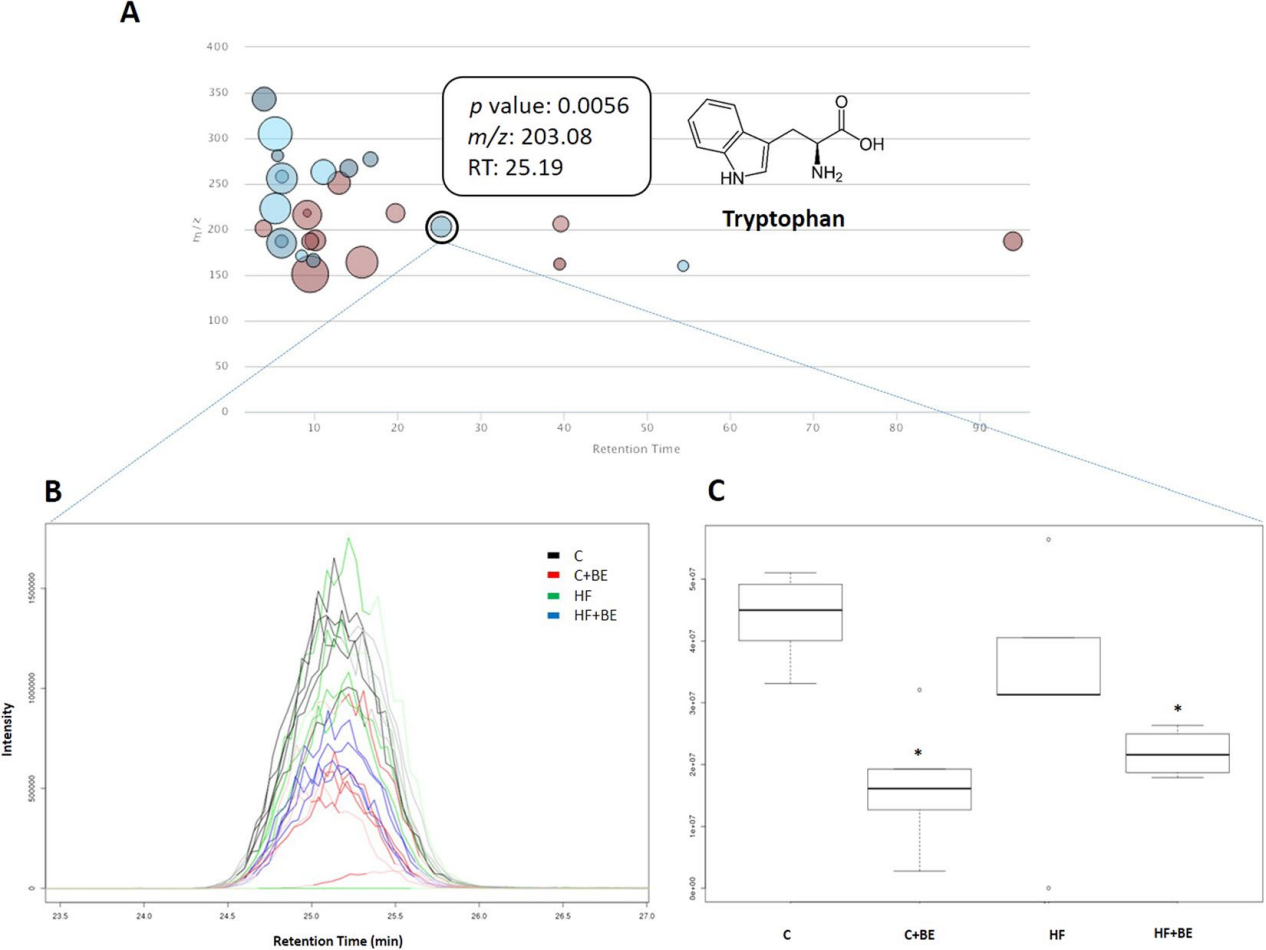
(**A**) Metabolite features whose level varies significantly (*p* < 0.01) across groups are projected on the cloud plot depending on their retention time (x-axis) and m/z (y-axis). Statistical significance (*p-*value) is represented by the bubble’s color intensity. The size of the bubble denotes feature intensity (only features with maximum intensity above 1 000 000 are displayed). Feature assignments (*p*-value, m/z, RT) are displayed in a pop-up window for the identified metabolite tryptophan. (**B**) Extracted ion chromatogram (EIC) and (**C**) Boxplot of tryptophan. *p < 0.05 vs respective control.

**Figure 6:**
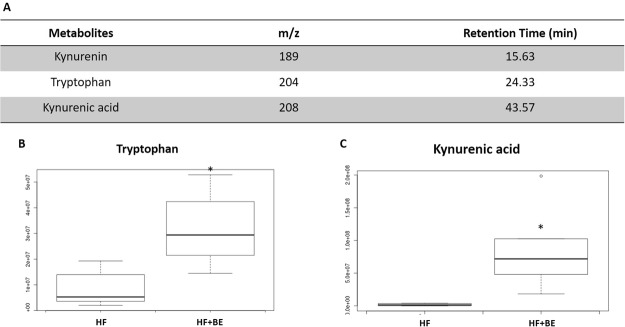
(**A**) Tryptophan and tryptophan metabolites searched in the urine of the animals of all groups. (**B**) Boxplot of tryptophan and (**C**) Boxplot of kynurenic acid. *p < 0.05 vs respective control.

The liquid chromatography-mass spectrometry (LC-MS) analysis revealed the existence of additional compounds in the animals’ fecal samples. Twenty-six compounds (with a maximum intensity above 1,000,000) were observed to be significantly altered between samples (*p* < 0.01) (Fig. 5). Tryptophan (one of the identified metabolites) was decreased in the groups supplemented with BE (*p* < 0.05, Fig. 5). Interestingly, fecal concentrations of tryptophan were positively correlated with TCK-1 expression in the hippocampus (*r* = 0.733, *p* < 0.05), indicating that changes in the gut bacterial genera prompted by BE could have altered host tryptophan metabolism. To verify this assumption, tryptophan and tryptophan metabolites (kynurenine and kynurenic acid) were searched in the urine of all animals. Tryptophan and kynurenic acid (Fig. 6) were increased in the urine of the animals fed with HF diet and supplemented with BE (*p* < 0.05), while kynurenine levels remained unchanged. On the contrary, no changes were observed in the animals of C + BE group.

## Discussion

The present findings demonstrate for the first time that blackberry anthocyanins are able to prevent some of the features of HF diet-induced dysbiosis. In addition, we demonstrate that anthocyanin-induced changes in the gut microbiota composition are related with their anti-neuroinflammatory properties. Finally, we propose that anthocyanins are able to counteract diet-induced neuroinflammation through the stimulation of tryptophan metabolism along the kynurenine pathway.

A number of studies have shown that mental illness may have origins in the gut^17–19^. Indeed, compelled by the study of Bruce-Keller *et al*., we have already proposed that HF diet-induced dysbiosis could be the trigger of neuroinflammation, a common hallmark of neuropsychiatry disorders^7^.

Besides changing the gut microbiota composition (HF-diet induced dysbiosis), HF diets can also increase intestinal permeability, facilitating the passage of LPS into the circulation^20^. In addition, LPS transport from the intestine towards target tissues can also be enabled by the chylomicrons synthesized in response to fat feeding^21^. Once in circulation, LPS may activate CD14/TLR4 signaling in target tissues, including the brain and can also disrupt the blood-brain barrier and induce neuroinflammation^22^. In our study, plasma LPS levels in animals fed with HF diet were not altered (data not shown), probably because the animals were fasted before sacrifice^23^. However, the propensity for increased fecal LPS levels may reflect the alterations in the gut microbiota composition that HF diet brought about and might be indicative of higher amounts of LPS in plasma. On the other hand, increased fecal LPS levels may activate dendritic cells within the intestinal barrier, increasing the production of cytokines and, thus contributing to peripheral and systemic inflammation^24^. In the present study, we evaluated whether anthocyanins were able to counteract these effects of HF diet.

The bioavailability of anthocyanins is considered to be low, therefore, it is expected that high amounts of these compounds reach the colon after consumption of anthocyanins-rich foods^14^. In fact, anthocyanins are extensively metabolized by the gut bacteria and these compounds can predominate during several days in the organism given the enterohepatic recirculation^14,25^. Concurrently, they are utilized by these microorganisms, and anthocyanins have been shown to selectively stimulate the growth of some bacterial groups^14^. Hidalgo *et al*. analyzed the effects of anthocyanins upon gut microbiota composition by real-time PCR, using batch culture fermentations. The authors showed that anthocyanins significantly enhanced the growth of *Lactobacillus-Enterococcus spp* and *Bifidobacterium spp*^26^. Here, we show for the first time a complete picture of the modulation of gut microbial genera by blackberry anthocyanins, in a rodent model.

BE was able to prevent some of the features of HF-diet induced dysbiosis, including fecal LPS levels. In the context of a standard diet, BE increased *Pseudoflavonifractor*. Previously, *Pseudoflavonifractor* was found to be decreased in long-term users of proton pump inhibitors which have been associated with increased risk of *Clostridium difficile* infection^27^. In addition, it has been suggested that increases in *Pseudoflavonifractor* may increase the success of obese patients in losing weight consistently^28^. In the context of HF diet, BE increased *Sporobacter* similarly to Goji berries that ameliorated chronic colitis through the promotion of beneficial gut bacteria^29^.

After showing that BE modulates the gut microbiota composition and prevent some of the features of HF-diet induced dysbiosis, we demonstrated that BE-induced changes in gut microbiota profile were correlated with BE anti-neuroinflammatory properties, previously assessed on the hippocampus of the same animals. BE consumption decreased TCK-1 expression in rat’s hippocampus whereas increased *Pseudoflavonifractor* and *Sporobacter*, in the context of C and HF diet, respectively. Although correlations do not always point towards causation, this reinforces the idea that BE protection against neuroinflammation can be due to gut microbiota modifications.

Besides LPS, other pathways may be involved in the bilateral communication between the gut and the brain^30^. Since tryptophan was decreased in the fecal samples of the animals supplemented with BE, we decided to explore the metabolic pathway of tryptophan. As a precursor of serotonin and kynurenine, changes in the supply and availability of this essential amino acid can have many implications for CNS functioning^31^. The dietary protein intake was the same in the groups supplemented with BE compared to the respective controls. Therefore, if tryptophan was decreased in the fecal samples of the animals supplemented with BE, it could be because BE was stimulating either the microbial catabolism of tryptophan or the host tryptophan metabolism. Around 90% of tryptophan is metabolized by the kynurenine pathway^32^. In this regard, we looked for kynurenine and kynurenic acid (tryptophan metabolites produced along this pathway) in the urine of all animals. Interestingly, we found that tryptophan and kynurenic acid were increased in the HF + BE group. This result suggests that tryptophan is being converted in kynurenine and, in turn, kynurenine is being converted into kynurenic acid which may act as an antagonist at excitatory amino acid receptors and has been implicated in major psychiatric diseases^33^. Alterations in the gut microbial composition might result in changes in serum and urine kynurenic acid levels and could thus modify CNS excitation and behavior^30^. Some studies have also reported the anti-inflammatory properties of kynurenic acid^34,35^.

The increased production in kynurenic acid could explain, at least in part, the anti-neuroinflammatory properties of anthocyanins, especially in the context of HF diet.

Furthermore, tryptophan could be also be utilized in serotonin synthesis in the gut^36^ or for indole production by gut bacteria which has been shown to increase intestinal barrier integrity^37^.

Nevertheless, a direct role of anthocyanins in the brain should not be ruled out. Anthocyanin metabolites can accumulate in the brain and exert their effects directly, including the stimulation of fractalkine secretion^12,38^.

In conclusion, anthocyanins alter host tryptophan metabolism, generating metabolites responsible for the control of CNS inflammation. This might be a possible mechanism behind their anti-neuroinflammatory properties. Anthocyanins may act, therefore, as mediators of the microbiota-gut-brain axis, allowing the control of neuroinflammation by gut microbiota modulation.

These results strongly suggest that dietary manipulation of the gut microbiota by anthocyanins could attenuate the neurologic complications of obesity. Anthocyanins emerge, therefore, as a new class of psychobiotics.

Lastly, these preclinical studies have prompted interest in whether targeting the gut microbiota with anthocyanins might be a viable strategy to influence tryptophan availability for kynurenine metabolism and neuroinflammatory control within CNS.

## Experimental Procedures

### Animals

Twenty-four male Wistar rats were randomly divided into four groups (n = 6 per group), as previously described: (C) standard diet; (C + BE) standard diet + blackberry anthocyanin rich extract; (HF) high-fat diet; (HF + BE) high-fat diet + blackberry anthocyanin rich extract^13,39^. Animals were fed ad libitum with “standard” (Teklad 2014, Harlan Laboratories, Santiga, Spain) or “high-fat” diets (D1245 Research Diets, New Brunswick, USA) for 17 weeks. Blackberry anthocyanin rich extract (BE, 25 mg/kg body weight/day) was obtained as previously described^13^. Briefly, blackberry anthocyanins (Rubus fruticosus) were extracted with 50% aqueous ethanol (pH 1.5, acidified with HCl) for 24 h at 22 °C. The solution obtained was filtered (50-μmnylon membrane) and concentrated using a rotary evaporator under at 30 °C. The concentrated extract was added to a polyamide gel column (mesh 100–120) to remove sugars. The sugar-free anthocyanin extract was freeze-dried and stored at −20 °C. BE was dissolved daily in sterile water and embedded in food pellets that animals had daily access to. Animal handling and housing protocols followed European Union guidelines (86/609/EEC) for the use of experimental animals. The study obtained ethical approval from the Ethical Committee of the Faculty of Medicine of University of Porto.

### DNA extraction from stool and 16S rRNA sequence analysis

Fresh fecal samples were collected directly from the colon of all animals, snap-frozen in liquid nitrogen and stored at −80 °C until further analysis. Genomic DNA was extracted and purified from stool samples using NZY Tissue gDNA Isolation Kit (NZYTech, Lisbon, Portugal) as previously described by Marques *et al*.^23^. Firmicutes and Bacteroidetes were quantified by real-time PCR as previously described by Marques *et al*.^23^. Libraries were prepared following the 16S Metagenomic Sequencing Library Preparation protocol from illumina (illumina; San Diego, CA, USA). The region of interest was captured using the Klidnworth *et al*. set of primers that covered the hypervariable region V3–V4 of the bacterial 16s rRNA^40^. Samples were pooled and loaded into the illumina MiSeq System and, then, sequenced using a 300PE combination according to manufacturer’s specifications.

Raw sequencing reads were merged with PEAR v0.9.6. Amplification primers were trimmed from the sequences obtained using the default program settings of cutadapt v1.9.1^41^. Sequencing quality filtering was subsequently applied to isolate the sequences having more than 300 nts with a mean quality score ≥20. Sequences were excluded from all downstream analyses. Sequences were also inspected for PCR chimera constructs. The resulting sequence reads were clustered into operational taxonomic units (OTU) at 98% similarity, using cd-hit program. NCBI database was used to assign a taxonomic classification to each read in the representative set. Reads with no hits in the reference sequence collection were classified as “null”. OTUs with a relative abundance <1% in all samples were considered non-significant and are not presented. Shannon’s richness index was calculated using the formula described in^42^.

### Neuroinflammation assessment

As previously described, neuroinflammatory markers were measured in the hippocampus of all animals using a predefined cytokine glass-based array (Quantibody Rat Cytokine Array; RayBiotech), according to manufacturer’s instructions^13^.

### Fecal LPS quantification

Quantification of LPS was performed using the Chromo-Limulus Amebocyte Lysate (Chromo-LAL) reagent (Associates of Cape Cod, Inc., Falmouth, MA, USA). Briefly, 1 mL of sterile saline solution (NaCl 0.9%) was added to 100 mg feces, vortexed and centrifuged (10 min, 10000 *g*, 4 °C) twice. Total supernatant (fecal water) was filtered with 0.45 μm filter and then with 0.22 μm filter. Fecal water and Chromo-LAL (1:1) were incubated at 37 °C for 20 min and absorbance was read every 10 s at 405 nm.

### Fecal and urine metabolome analysis by HPLC/Orbitrap

The fecal water obtained for LPS quantification was also used for metabolomics experiments. Urine samples were prepared according to the procedure described by Marques *et al*.^43^. Samples were analyzed by HPLC/Orbitrap according to the method described by Fernandes *et al*.^44^. Mass spectrometry (MS) data was uploaded into XCMS Online and was processed as a multi-group experiment using the default HPLC/Orbitrap parameters in negative mode (fecal samples) or positive mode (urine samples)^45^. Isotopes and adducts were annotated using CAMERA and arranged into feature groups. Metabolite features were selected to assess the differences between the fecal and urine samples of C, C + BE, HF and HF + BE groups. Tryptophan, kynurenine and kynurenic acid (MilliporeSigma, St. Louis, MO, USA) were used as standards.

### Statistical analysis

Two-way ANOVA was used to determine the main effects of diet (CDe vs HF diet), BE supplementation (No BE vs BE) and their interaction. In XCMS online, one-way ANOVA followed by a post hoc multi comparison test was used in multi group experiment. To compare the differences between two groups, *t*-test was used. Correlation between variables was established using Spearman’s correlation test. Statistical analyses were performed using SPSS Statistics 23 (IBM, USA) software. Differences were considered statistically significant when *p* < 0.05. Principal component analysis (PCoA) was performed in R 3.0.2 (The R Foundation, New Zealand) with the RStudio 0.97.310 package. Heatmaps were elaborated using CIMminer platform (https://discover.nci.nih.gov/cimminer/home.do).
